# Evaluation of the impact of high altitude on pre-hospital anaesthetic administration: a scoping review

**DOI:** 10.1016/j.bjao.2026.100643

**Published:** 2026-08-24

**Authors:** Olivia G. Hobrough, Stuart W. Reilly, Owen W. Tomlinson

**Affiliations:** 1 University of Exeter Medical School, Exeter, UK; 2 Department of Anaesthesia, Worcestershire Acute Hospitals NHS Trust, Worcestershire, UK

**Keywords:** anaesthesia, analgesia, expedition medicine, high-altitude, pre-hospital, review

## Abstract

**Background:**

Increasing high-altitude travel and habitation drive demand for care in these environments. High altitude presents unique challenges to anaesthetic administration as hypobaric hypoxia affects physiology, pharmacology, and equipment performance, yet no guidelines exist. This scoping review aimed to identify anaesthetics used at HA, evaluate the impact of high altitude on anaesthetic use, and highlight research gaps.

**Methods:**

This review followed Arksey and O’Malley’s methodological framework and is reported according to the Preferred Reporting Items for Systematic Reviews and Meta-Analyses extension for Scoping Reviews guidelines. MEDLINE, Embase, and Web of Science were searched (October 2024 to May 2025). English-language studies, published after 1980, involving human participants receiving any anaesthetic agent above 1500 m were included. Critical appraisal is undertaken using Critical Appraisal Skills Programme checklists.

**Results:**

A total of 21 articles (14 original studies, three case reports/series, and four letters to the editor; *n*=3162) were included. Mean study altitude was 2922 m (95% confidence interval [CI] 2412–3432), with a range of 1610–4500 m. Participants were predominantly female (78.9%) with a mean age of 20.7 years. CIs for sex distribution and age could not be calculated because participant-level variance data were not consistently reported. Techniques included general, neuraxial, analgesic, and local modalities. Inhalational agents showed unreliable performance, whereas propofol-fentanyl and ketamine were safe when titrated. Neuraxial anaesthesia required higher doses. Methoxyflurane was effective. Sensitivity to nitrous oxide, opioids, and benzodiazepines seems increased. Lidocaine half-life was prolonged.

**Conclusions:**

Anaesthesia at high altitude remains complex and high-risk. Evidence indicates general anaesthesia should be avoided where possible; Total i.v. anaesthesia may be a practical option where general anaesthesia is required, although the available evidence is limited. Neuraxial techniques are effective in-hospital but impractical pre-hospital. Methoxyflurane and ketamine seem promising for field use at high altitude, whereas nitrous oxide, opioids, and benzodiazepines may carry increased risks. Altitude-specific research and expert consensus are urgently required.

**Editor’s key points**

• High-altitude changes the safety and effectiveness of anaesthesia delivery.
• This review provides a comprehensive summary of the available evidence on anaesthesia at high altitude.
• Ketamine and methoxyflurane seem to be the most promising options for pre-hospital care at high altitude.
• Inhalational anaesthetics, nitrous oxide, opioids, and benzodiazepines require extra caution because altitude may alter their effects.
• More high-quality research is urgently needed to develop evidence-based guidelines for anaesthetic practice at high altitude.

## Rationale

Accessibility and demand for adventure travel have dramatically increased the number of travellers to high-altitude. For example, between 2003 and 2012, visitors to Kilimanjaro increased from 28 000 to 56 000.^1^ Similarly, the number of trekkers/mountaineers visiting Nepal increased from 69 619 in 1993 to 187 682 in 2018,^2^ with “traffic jams” on Everest being heavily publicised.^3^

In parallel, permanent habitation at high altitude is also increasing, with an estimated 86.6 million people living above 2500 m in 2021 compared with 83.0 million in 2010.^4^ Consequently, the demand for medical care at high altitude is increasing.

Definitions of high altitude vary. The Wilderness Medicine Society defines it as >2500 m, as symptoms of altitude-related illness typically occur above this elevation.^5^ However, as basic respiratory and cardiovascular adaptations, such as tachypnoea and tachycardia,^6^ begin to occur at approximately 1500 m,^7^, ^8^, ^9^ the more conservative definition of 1500 m is used throughout this review. This ensures that important data are not excluded.

These adaptations occur at high altitude owing to decreased oxygen availability with decreasing barometric pressure.^9^ Thus, being at high altitude can be fatal in itself.^8^ Furthermore, other factors, including severe cold, dehydration, high winds, solar radiation, and recreational activities, such as skiing, climbing, and mountaineering, also increase morbidity.^7^ In these settings, access to definitive medical care is often delayed, making the pre-hospital period (defined as the time before reaching definitive care^10^), crucial for positive clinical outcomes.^11^^,^^12^ In high-income countries, 15%–32% of trauma deaths (at varying altitudes) are considered preventable with good pre-hospital management.^13^^,^^14^ This preventable number would likely increase in remote high altitude regions, emphasising the need for focus in this area. Therefore, we consider both pre- and in-hospital anaesthesia throughout this review.

Anaesthetics, defined as any drug or substance that causes a loss of feeling or awareness,^15^ are central to effective medical care.^10^ They are sub-categorised by method of use. The standard methods of anaesthesia are analgesia/sedation, local anaesthesia, regional anaesthesia, and general anaesthesia.^16^ However, the effects of hypobaric hypoxia on human physiology, pharmacology, and equipment performance complicate pre-hospital administration.^17^

Interest in anaesthesia at high altitude dates to 1933, when chloroform was administered at approximately 4250 m on an Everest expedition.^18^ The patient’s subsequent cardiopulmonary arrest has long been attributed to the effects of altitude on anaesthetic delivery. Nearly 100 yr on, understanding of the effects of high altitude on anaesthetics remains limited, and no guidelines exist for their use.^19^ Therefore, this review lends itself to a scoping approach to present a broad overview of the existing literature to inform best practice.

Based on the known physiological effects of hypobaric hypoxia on human function, pharmacology, and anaesthetic equipment performance, we hypothesised that high altitude would influence the administration, efficacy, and safety of multiple anaesthetic techniques and agents. We further hypothesised that the available evidence would be limited and heterogeneous, reflecting the logistical and ethical challenges of conducting research in HA environments.

## Objectives

This scoping review aimed to systematically synthesise the existing information on the influence of high altitude upon anaesthetic administration. In particular, it sought to determine which anaesthetics are used in high altitude environments and how, clarify the impact of high altitude on anaesthetic use, and identify research gaps to inform and encourage future studies.

Given the anticipated heterogeneity in the literature, making formal clinical recommendations is beyond the scope of this type of review.

## Methods

### Ethics and reporting standards

Ethical approval was not required for this study as it is a scoping review of previously published literature and does not include identifiable data.

This review was conducted and reported in accordance with the Preferred Reporting Items for Systematic Reviews and Meta-Analyses extension for Scoping Reviews (PRISMA-ScR) checklist.^20^

The review followed Arksey and O’Malley’s six-stage methodological framework.^21^ The research protocol is available on the Open Science Framework.^22^, ^23^

#### Stage 1: identifying the research question

A broad research question was identified, using the “population, concept, context” (PCC) framework, to capture a wide range of publications relevant to anaesthetic administration at high altitude. The PCC framework is recommended by the Joanna Briggs Institute^24^ and endorsed by PRISMA-ScR to identify the main concepts for review, which in turn help to inform the search strategy (Supplementary File 1).

The research question was: "what evidence exists on the administration, effectiveness, safety, and practical considerations of anaesthetic agents and techniques in humans at high altitude?”

#### Stage 2: identifying relevant studies

Initial scoping searches informed the development of key terms common to appropriate literature. This in turn developed search concepts tables, which considered a wide range of keywords and Medical Subject Headings (MeSH) that can be used to describe HA anaesthetic administration.

Search testing was performed for searches conducted in Medical Literature Analysis and Retrieval System Online (MEDLINE), Excerpta Medica Database (Embase), and Web of Science. These databases were selected to ensure comprehensive coverage of the available literature. MEDLINE provides extensive clinical data, Embase adds broader pharmacological and international conference content, and Web of Science captures interdisciplinary and citation-linked studies. Search testing involved reviewing the MeSH terms used to tag three seed articles and adapting the search so that these were always included. Backwards searching of these three seed articles also identified relevant articles not found in the preliminary search, so further refinement of the MeSH terms and keywords was made.

MeSH terms were available in MEDLINE and Embase and were varied to reflect the vocabulary of each database (Supplementary File 2). Web of Science does not have a controlled vocabulary system, so to enhance the search, additional synonyms and spellings were used, including the brand names of anaesthetics (Supplementary File 2).

Forwards and backwards searches were then conducted to identify any additional articles not captured by the initial search.

#### Stage 3: study selection

In keeping with the rapid scoping approach adopted for this review, the identified studies were screened by a single author (OH) from October 2024 to May 2025. This approach was chosen to facilitate timely synthesis of a broad evidence base. Pilot searches, to refine the above strategy, were conducted between 1st October 2024 and 1st March 2025. Final searches for MEDLINE, Embase and Web of Science were completed by April 14th 2025. Study selection, and forward/backwards citation searches on the final included studies, was completed by May 1st 2025. A second reviewer (OT) independently reviewed all studies for which eligibility was uncertain and resolved any queries through discussion with the primary reviewer until consensus was reached. Titles and abstracts were screened against the inclusion and exclusion criteria, again developed using the PCC framework^24^ (Supplementary File 1). Letters to the editor were included, as they are a recognised way to report findings anecdotally where high-quality research is difficult to undertake.

#### Stage 4: data charting

Full-text screening was conducted and data extracted by a single author using Google Sheets to collect specific details relevant to the research questions (Supplementary File 3):

1. Title
2. Authors
3. Article type
4. Publication year
5. Study numbers (if applicable)
6. Study location
7. Study aims
8. Sample population: native lowlanders *vs* highlanders (if applicable)
9. Level of altitude in metres
10. Anaesthetic sub-category
11. Anaesthetic agents
12. Clinical recommendations/conclusions from the paper

The extraction form was developed *a priori* and refined iteratively during the review process to capture emerging variables relevant to the research question.

#### Stage 5: data synthesis

Basic analysis of key data characteristics is presented in tabular form (Table 1) and in Figure 2.

**Table 1:** Participant characteristics within 21 included articles. ∗Letters to the editor do not detail study participants, so the table only includes data from 17 of the included 21 articles.

Parameter (number of studies)	Number
**Article type (*n*=21)**	
Original research	14
Case report/series	3
Letter to the editor	4
**Sample size (*n*=17∗/21)**	
Pooled	3162
Range	1–1856
**Sex of participants (*n*=17∗/21)**	
Male	592 (21.1%)
Female	2218 (78.9%)
Undocumented (*n*=3)	
**Age of participants, year (*n*=17∗/21)**	
Mean	20.7
Range	0–61
**Sample populations (*n*=17∗/21)**	
Native lowlander	373 (40.9%)
Native highlander	538 (59.1%)
Undocumented (*n*=2)	
**Special populations (*n*=17∗/21)**	
Pre-hospital (*n*=4)	41
In-hospital (*n*=13)	3121
○ Non-pregnant adults (*n*=8)	484
○ Pregnant adults (*n*=3)	1966
○ Paediatrics (*n*=2)	671
**Study altitude, metres (m) (*n*=17∗/21)**	
Mean of maximum altitude	2922
Range of maximum altitude	1610–4500
Comparison study (*n*=8)	0 *vs* 1646
	400 *vs* 2200–4500
	31 *vs* 1890
	150 *vs* 3399
	304 *vs* 3505
	171 *vs* 3000
	0 *vs* 1460 *vs* 3300
	0–1700
**Altitude type (*n*=17∗)**	
Real-world	16
Simulated	1 (0 m comparedwith 1460 and3300 m)
**Study location by country (*n*=17∗)**	
Nepal	4
China	2
Austria	2
USA	2
Colombia	1
Peru	1
South Africa	1
Zimbabwe	1
India	1
Turkey	1
Saudi Arabia	1

Critical appraisal of the data was undertaken by the first author (OH) using the Critical Appraisal Skills Programme (CASP) checklists.^25^ For each included article, the study design was identified, and the most appropriate CASP checklist applied (Supplementary File 4). For letters, case reports, and case series, CASP does not provide dedicated checklists; these are appraised narratively only. Given the heterogeneity in study designs and the corresponding CASP checklists, no formal scores are reported. Instead, methodological strengths, limitations, and uncertainties were summarised descriptively in line with the CASP recommendations.^25^ These were then independently reviewed by a second author (SR). Given the scoping review methodology, studies were not excluded based on methodological quality. Instead, narrative CASP appraisal findings were used to contextualise the strengths and limitations of the conclusions, in Table 2.^26^, ^27^, ^28^, ^29^, ^30^, ^31^, ^32^, ^33^, ^34^, ^35^, ^36^, ^37^, ^38^, ^39^, ^40^, ^41^, ^42^, ^43^, ^44^, ^45^

**Table 2:** Summary of reported conclusions. BIS, bispectral index; GRADE, Grading of Recommendations, Assessment, Development and Evaluation; HA, high altitude; N/A, not available.

Anaesthetic type	Conclusions	Comments
**General**	Delivery and monitoring of inhalational anaesthetics may be unreliable at high altitude.	Consistent observational reasoning^26^, ^27^, ^28^; high potential harm if not followed.
	Propofol-fentanyl anaesthesia, titrated to Bispectral Index, may be safe at high altitude.	Supported by one small, controlled study^29^; physiologically plausible. Requires monitoring
	Ketamine may be suitable in resource-limited environments, but the availability of simple ventilatory equipment and the skills to use them are mandatory.	Consistent across multiple studies^30^^,^^41^^,^^42^; physiologically plausible; requires airway equipment
**Regional**	Neuraxial anaesthesia with bupivacaine requires higher doses and has a slower onset at high altitude.	Consistent findings across small studies^31^^,^^32^; physiologically plausible
	There is no advantage for epidural ropivacaine 0.2% over bupivacaine 0.125%.	Evidence from single comparative study^33^
**Analgesia/sedation**	Nitrous oxide efficacy is inconsistent at high altitude.	Evidence from multiple studies^34^, ^35^, ^36^
	Methoxyflurane is effective in pre-hospital high altitude environments.	Evidence from an observational study and case report demonstrate consistent efficacy^38^^,^^39^; practical in pre-hospital environments^37^
	Ketamine can be used safely for analgesia at high altitude; careful titration is required, especially for lowlanders. Airway equipment and skills should be mandatory.	Supported by multiple studies^31^^,^^41^^,^^42^; consistent efficacy, but dosing variability and potential safety risks; requires airway equipment. Caution is required crossing borders.^40^
	Caution and dose reduction are required when using opioids at high altitude.	Single observational study suggests reduced opioid requirements at high altitude^42^; significant safety risks
	Benzodiazepines are unlikely to be safe in pre-hospital high altitude settings.	RCT evidence of ventilatory depression at high altitude even with low doses^44^; physiologically plausible; significant safety risks
**Local**	No conclusions can be drawn for the use of local anaesthetics at high altitude.	Single pharmacokinetic study^45^; insufficient clinical outcome data

There is no meta-analysis because the collected data were anticipated to be highly heterogeneous.

### Data availability

Data extracted for this review are presented in the Supplementary Files.

## Results

### Article selection

**Fig 1:**
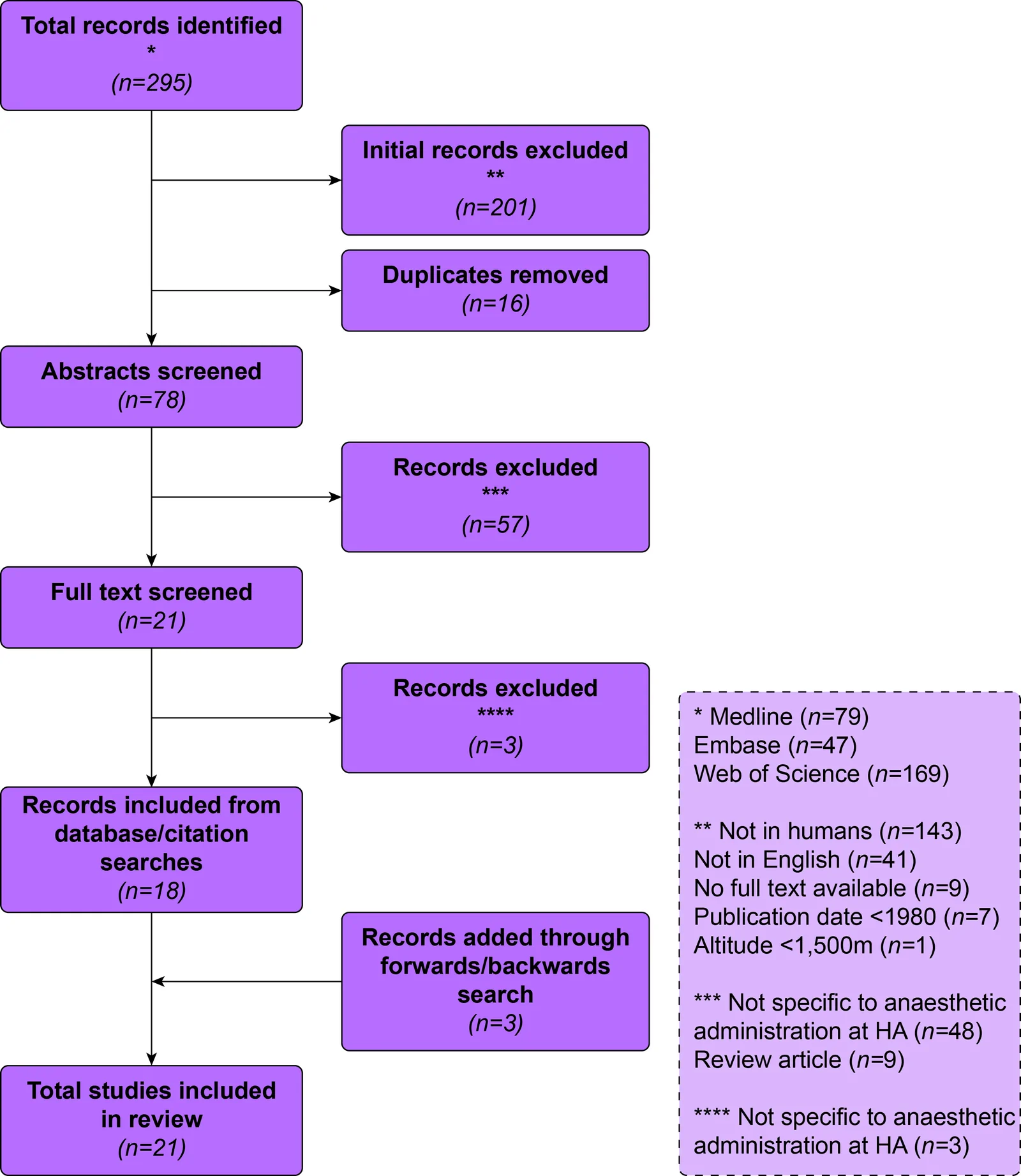
Preferred Reporting Items for Systematic Reviews and Meta-Analyses flow diagram for this scoping review on anaesthetic administration at high altitude

The initial database searches yielded 295 results, of which 18 were included. A further three studies were identified through backwards/forwards searching of the original results; a total of 21 records were reviewed (Fig. 1).

The small number of eligible studies highlights the limited research activity in anaesthetic practice at high altitude. Several factors potentially contribute, but logistical and ethical challenges are likely the primary barriers to controlled or large-scale studies.

### Data characteristics

Data characteristics of the 21 included studies are presented in Table 1. All studies are detailed in Supplementary File 3.

Of the 21 included articles, 14 were original research, two were case reports, one was a case series, and four were letters to the editor. Of the 14 pieces of original research, there was one up-down sequential study, one open-label study, three retrospective observational studies, one prospective randomised controlled study, three prospective controlled interventional studies, one prospective controlled comparative study, one randomised, double-blind, placebo-controlled trial, one prospective cohort study, and one prospective observational study.

The sample size ranged across the 21 articles from zero subjects in the “letters to the editor” (*n*=4) to 1–1856 subjects in the case reports, series, and original research (*n*=17), with a pooled sample size of 3162 subjects. The mean sample size of the articles that included participants was 186 subjects, with a median of 50 subjects. Excluding three articles that did not report gender, studies reported a female predominance of 78.9%. This is caused, in part, by three articles evaluating the effect of altitude on anaesthesia in pregnancy/delivery (*n*=3), which together account for a pooled sample size of 1966 subjects. The mean age of the participants was 20.7 years with a range of 0–61 years. Two studies were specifically related to children <18 years. Confidence intervals (CIs) for sex distribution and age could not be calculated because participant-level variance data were not consistently reported.

The maximum altitude considered in any study ranged from 1610 to 4500 m, with a mean of 2922 m (95% CI 2412–3432), across countries. One article used a hypobaric chamber to simulate high altitude.^26^ This is helpful in overcoming logistical, ethical, and safety challenges of field research, but limits external validity, as hypobaric chambers fail to account for other interacting physiological stressors, including cold, dehydration, fatigue, and prolonged hypoxia.

The profession of the person administering the anaesthetic is not specifically reported by any of the studies. This omission limits the interpretation of the safety, efficacy, and applicability of the reported outcomes; without clarity on the provider’s background, it is difficult to assess the feasibility of replicating the reported techniques in comparable settings or to develop appropriate training recommendations.

### Anaesthetics

**Fig 2:**
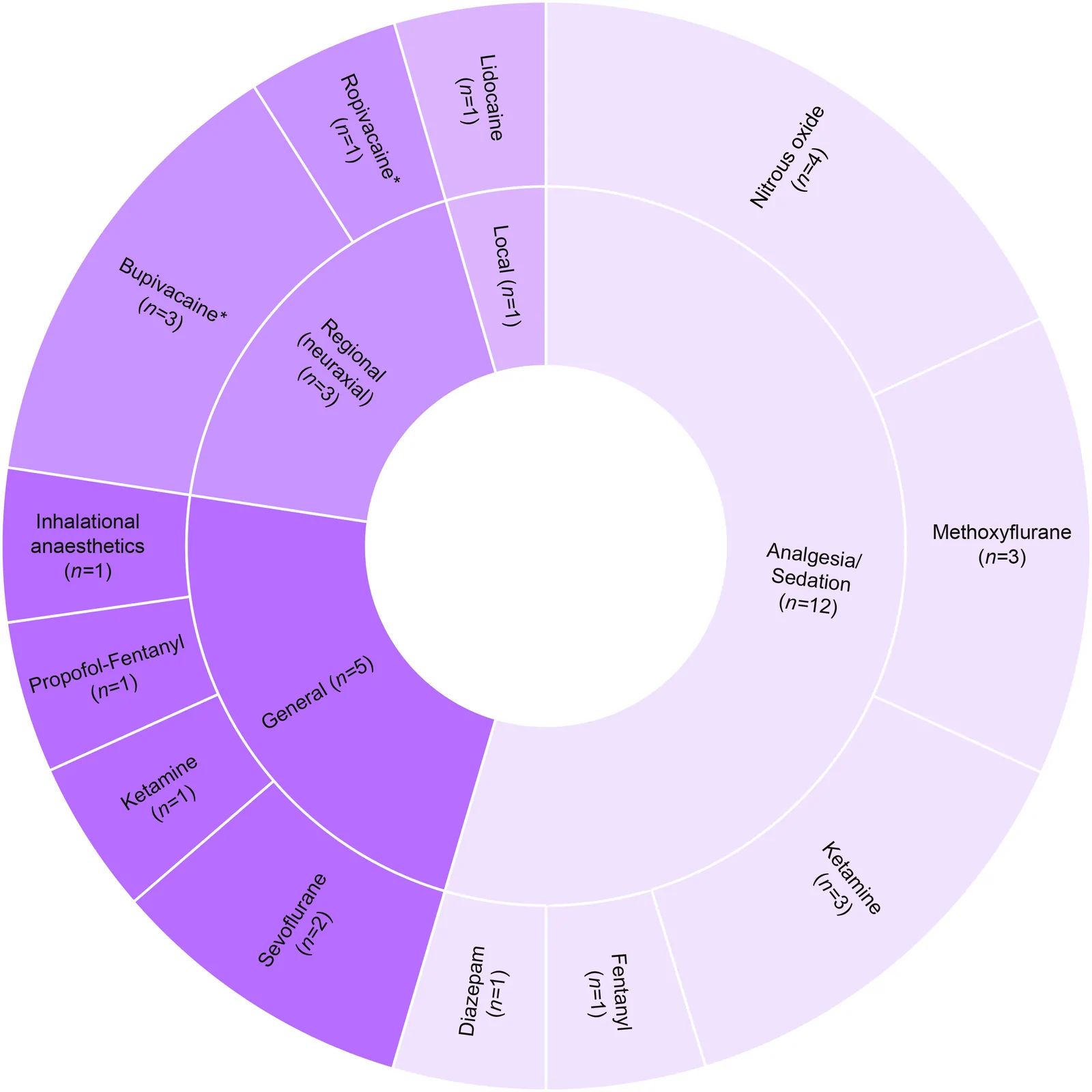
Sunburst chart illustrating the distribution of anaesthetic techniques and agents identified in the included literature. The inner ring represents the anaesthetic categories, whereas the outer ring represents individual anaesthetic agents. Segment size is proportional to the frequency with which each category or agent was reported across the 21 included studies. *∗*Includes comparison study.

The included articles indicate that a range of anaesthetic agents have been used at high altitude (Fig. 2). Results are summarised by type of anaesthesia. An overall narrative summary of findings table (Table 2^26^, ^27^, ^28^, ^29^, ^30^, ^31^, ^32^, ^33^, ^34^, ^35^, ^36^, ^37^, ^38^, ^39^, ^40^, ^41^, ^42^, ^43^, ^44^, ^45^) is presented in the Main Findings of the Discussion Section CASP critical appraisal of each of the included articles is presented in Supplementary File 4.

### General anaesthetic

Five articles addressed general anaesthetic administration at high altitude.^26^, ^27^, ^28^, ^29^, ^30^ Three focused on inhalational anaesthetics, highlighting the impact of reduced barometric pressure on the partial pressure and delivery of volatile agents and their potential to suppress respiratory drive and exacerbate hypoxia.^26^, ^27^, ^28^ They emphasise the subsequent importance of accurately monitoring the depth of anaesthesia using either mean alveolar concentration (MAC) or bispectral index (BIS). Giraldo and colleagues^25^ conducted a prospective cohort study at 2600 m and found a higher-than-expected incidence of anaesthetic overdose when relying on MAC, concluding that BIS is a more reliable alternative in HA environments. In response, Karim^26^ highlighted the sensitivity of MAC to variables such as age and emphasised the need for further research to clarify the altitude-MACage relationship. Baumgarten^27^ proposed using closed-circuit anaesthesia to mitigate the limitations of MAC at altitude.

The other two articles investigated total i.v. anaesthesia (TIVA). Puri and colleagues^28^ conducted a prospective interventional study that demonstrated that high altitude residents required significantly higher induction and maintenance doses of propofol-fentanyl than lowlanders, although anaesthesia remained safe when titrated to BIS. Maharjan^29^ reported the successful use of ketamine without intubation for cleft lip surgery in children at altitude, provided that appropriate monitoring and airway equipment were available.

### Regional anaesthetic

Three articles addressed neuraxial anaesthesia at high altitude.^31^^,^^32^^,^^46^ Overall, the findings suggest altered pharmacodynamics of bupivacaine at altitude, attributed to increased CSF volume. Both Yang and colleagues^30^ and Aksoy and colleagues^46^ observed higher epidural dose requirements and altered block characteristics at 2000 and 1890 m, respectively. Shokry and colleagues^31^ conducted an RCT comparing epidural ropivacaine 0.2% and bupivacaine 0.125% combined with fentanyl for labour analgesia at 2200 m. No significant difference in efficacy or safety was found between the two regimens.

### Analgesia/sedation

Thirteen articles addressed analgesia/sedation at high altitude, including nitrous oxide (N_2_O), methoxyflurane, ketamine, opioids, and benzodiazepines.

#### Nitrous oxide (N_2_O)

Four studies examined the use of N_2_O with mixed findings.^34^^,^^33^, ^34^, ^35^ Wood and colleagues^32^ found comparable efficacy between high altitude and low-altitude institutions, whereas James and colleagues^33^ and Nieto and colleagues^34^ both reported diminished analgesic effects at higher altitude. Cleaton-Jones and colleagues^35^ observed only subjective differences, with increased “tingling” reported by participants at 1700 m compared with sea level, but no statistically significant change in level of consciousness.

#### Methoxyflurane

Three articles examined the use of methoxyflurane at high altitude with consensus supporting its effectiveness in pre-hospital and remote environments.^37^^,^^38^^,^^47^ Despite theoretical concerns regarding reduced vaporisation at lower barometric pressures from Windsor and colleagues,^47^ evidence from both Wilkes and colleagues^47^ and Egger and colleagues^37^ indicates that methoxyflurane retains its efficacy even at elevations exceeding 4000 m. Egger and colleagues^37^ also demonstrated significant pain reduction with no adverse effects at moderate altitude.

#### Ketamine

Four studies addressed the use of ketamine at high altitude with consistent support for its utility in both pre-hospital and resource-limited settings.^30^^,^^39^, ^40^, ^41^ Across these varied study designs, ketamine was found to be effective even at very high altitudes (>3500 m), offering the advantage of maintaining cardiovascular stability and not requiring advanced airway equipment when used cautiously. However, concerns over inter-individual variability were noted, particularly with intranasal administration by Ellerton and colleagues.^38^ Grocott and colleagues^39^ also demonstrate that lower doses than those typically required at sea level were sufficient to achieve deep anaesthesia.

#### Opioids

Only one study explored opioid requirements at high altitude.^42^ This observational study identified a substantial reduction in the dose of fentanyl amongst patients residing at high altitude.^42^ However, no studies have clarified the reasons for this.

#### Benzodiazepines

Only one study investigated the use of benzodiazepines at high altitude.^43^ Röggla and colleagues^42^ confirmed the theoretical altitude-related vulnerability to the respiratory depressant effects of diazepam.

### Local anaesthetics

One article was identified concerning local anaesthetic administration at high altitude.^44^ Zhang and colleagues^43^ demonstrated a prolonged half-life of lidocaine in individuals living at high altitude. This may be linked to downregulation of hepatic CYP3A activity, as supported by animal data.

### Summary of findings

The conclusions of the included articles are presented in Table 2.^26^, ^27^, ^28^, ^29^, ^30^, ^31^, ^32^, ^33^, ^34^, ^35^, ^36^, ^37^, ^38^, ^39^, ^40^, ^41^, ^42^, ^43^, ^44^, ^45^ CASP critical appraisal of each article is presented in Supplementary File 4.

## Discussion

### Main findings

This review identified 21 articles investigating anaesthetic administration at high altitude, encompassing general, neuraxial, local, and analgesic techniques. Overall, the available evidence suggests that high altitude influences the pharmacokinetics, pharmacodynamics, and delivery of several anaesthetic agents, although the magnitude and direction of these effects vary between drug classes and clinical settings. Although methoxyflurane, ketamine, and carefully titrated TIVA showed favourable performance in selected settings, the evidence base remains limited and highly heterogeneous. Consequently, robust clinical recommendations cannot yet be made, highlighting the need for high-quality altitude-specific research.

General anaesthesia studies highlighted altered volatile agent delivery at reduced barometric pressures, alongside evidence that TIVA techniques may require dose adjustment at high altitude. Regional anaesthesia studies suggested altered neuraxial pharmacodynamics, with higher dose requirements and modified block characteristics reported. The largest body of literature concerned analgesia and sedation, particularly N_2_O, methoxyflurane, and ketamine. Findings for N_2_O were inconsistent, whereas methoxyflurane and ketamine demonstrated relative effectiveness in remote and pre-hospital high altitude environments. Limited evidence addressed opioids, benzodiazepines, and local anaesthetics, suggesting the need for opioid dose reduction, increased respiratory vulnerability with benzodiazepines, and prolonged lidocaine half-life. Collectively, these findings demonstrate considerable physiological and pharmacological variability across multiple anaesthetic approaches at high altitude. However, high altitude should not be considered an isolated variable but rather a complex environment characterised by interacting physiological and environmental stressors, including hypoxia, cold exposure, dehydration, physical exertion, and variable acclimatisation status.

Only 21 articles met the inclusion criteria, highlighting the limited evidence available in this field. Nevertheless, the breadth of the retrieved literature provides valuable insight into the clinical, physiological, pharmacological, and logistical challenges associated with anaesthetic administration at high altitude. Substantial heterogeneity across study designs, altitudes, anaesthetic agents, patient populations, and clinical settings limits direct comparison and meaningful synthesis of findings. This variability, together with the predominance of in-hospital studies, small sample sizes, and largely observational or anecdotal evidence, reduces the certainty and generalisability of the conclusions. Although in-hospital studies were included to provide important physiological and pharmacological context, their findings may not be directly applicable to pre-hospital environments. Furthermore, only one RCT was identified, limiting the overall methodological quality of the evidence.

Despite these limitations, several consistent themes emerged, underpinning the clinical insights presented in the table below (Table 3^7^^,^^18^^,^^29^, ^30^, ^31^^,^^34^^,^^35^^,^^37^, ^38^, ^39^, ^40^, ^41^^,^^43^^,^^47^, ^48^, ^49^, ^50^, ^51^, ^52^, ^53^, ^54^, ^55^, ^56^, ^57^, ^58^, ^59^, ^60^, ^61^). These should not be interpreted as formal clinical recommendations but rather as evidence-informed observations derived from the currently available literature. They provide a foundation for future research and could provide provisional recommendations that require validation and endorsement from qualified and experienced clinicians.

**Table 3:** Clinical insights for use of anaesthetics at high altitude. BIS, bispectral index; FERB, field-expedient regional block; GA, general anaesthetic; HA, high altitude; N/A, not available; N_2_O, nitrous oxide; TIVA, total i.v. anaesthesia.

Agent	What we already know	What this review adds
**General anaesthetics**	Pre-hospital GA is high-risk, especially at altitude.^18^^,^^48^^,^^49^ When pre-hospital GA is clinically indicated, it should adhere to in-hospital anaesthetic standards, including equivalent expertise, techniques, equipment, and monitoring as per the AAGBI.^78^	Inhalational anaesthetics are unreliable at high altitude.^7^ TIVA using propofol-fentanyl anaesthesia, titrated to BIS, can be used safely at high altitude.^29^ Ketamine may be a suitable alternative in low-resource environments, but the availability of simple ventilatory equipment and the skills to use them are vital.^30^
**Regional anaesthetics**	Neuraxial is the preferred method of in-hospital anaesthesia at high altitude owing to its minimal effect on ventilation and decreased risk of aspiration.^31^ Neuraxial anaesthetics are impractical in pre-hospital environments and should likely be avoided.^51^	Slower onset and higher doses are expected to achieve successful neuraxial blockade at high altitude.^31^^,^^50^ Bupivacaine is currently the recommended agent for high altitude neuraxial anaesthesia based on available evidence^31^; further comparative studies are needed. No research exists examining nerve blocks at high altitude to inform clinical recommendations. High-quality primary research of FERBs at high altitude would be extremely valuable.
**Analgesia/sedation**		
N_2_O	N_2_O is an effective analgesic^52^ but is associated with significant practical issues, including transport difficulties^51^ and instability at temperature extreme.^38^	Evidence for the efficacy of N_2_O at high altitude is mixed.^34^^,^^35^ Data are insufficient to support routine use.
Methoxyflurane	Methoxyflurane is a safe and effective pre-hospital analgesic.^53^^,^^56^	Methoxyflurane may demonstrate potential as a first-line analgesic for use at high altitude owing to its efficacy, practicality, and minimal side-effect profile.^37^^,^^38^^,^^47^ Methoxyflurane is contraindicated in patients with hepatic or renal impairment and those with a personal or family history of malignant hyperthermia.^54^^,^^55^ Methoxyflurane is not licensed for use in several countries and in children aged <18 yr.^57^ Check before travel.
Ketamine	Ketamine is commonly suggested as the analgesic agent of choice in the pre-hospital environment^58^ and at high altitude.^39^^,^^59^	Ketamine can be used safely for analgesia at high altitude with extreme caution.^30^^,^^40^^,^^41^ Careful titration is highly important, especially in lowland residents, as deep anaesthesia may be achieved with significantly lower doses than at sea level.^40^ The availability of airway equipment and the skills to use them is vital.^30^^,^^40^^,^^41^
Opioids	Opioids induce hypoxia by diminishing hypoxic ventilatory drive.^60^ The limited available evidence and the risk of ventilatory depression support cautious, closely monitored opioid use at high altitude; evidence is insufficient to determine safety in pre-hospital settings.	High-quality studies are required to demonstrate safety and efficacy of opioids at high altitude.
Benzodiazepines	Benzodiazepines commonly cause ventilatory depression.^61^	Evidence suggests increased risk with use of benzodiazepines in pre-hospital high altitude environments.^43^
**Local anaesthetics**	N/A	No clinical insights can be made for the use of local anaesthetics at high altitude.

### General anaesthetics

Inhalational anaesthetic gases are inherently unreliable at high altitude owing to reduced ambient partial pressures, requiring increased vapour delivery, whereas low ambient temperatures impair vaporisation.^7^^,^^62^ Therefore, monitoring anaesthetic depth, as considered by Giraldo and colleagues,^25^ Karim,^26^ and Baumgarten,^27^ is critical. Even at low altitude, overdosing prolongs recovery and increases respiratory complications, postoperative delirium, nausea, or vomiting,^45^ whereas underdosing risks intraoperative awareness.^25^

Current evidence from Giraldo and colleagues^25^ suggests that, at high altitude, BIS, which quantifies anaesthetic depth via EEG parameters, is superior to MAC, which is affected by barometric pressure changes.^63^ Baumgarten^27^ proposed that closed-circuit systems may mitigate MAC limitations, but this is theoretically unlikely as closed systems do not overcome changes in barometric pressure, so further high-quality studies are required to substantiate the claim.

TIVA may offer a viable alternative. Puri and colleagues^28^ concluded that propofol-fentanyl anaesthesia, titrated to BIS, is safe at high altitude, but that high altitude residents require significantly higher doses of propofol-fentanyl for both induction and maintenance than lowlanders. This may result from increased cardiac output compensating for hypoxia,^64^ which has been shown to increase propofol requirements.^65^ However, studies on cardiac output in highlanders *vs* lowlanders remain contradictory.^66^^,^^67^ Further studies are required to demonstrate causation. Maharjan^29^ similarly demonstrated that ketamine can also be used safely at high altitude with careful titration. Lowland residents seem particularly susceptible to deep anaesthesia at reduced doses, emphasising the need for cautious administration.^30^^,^^40^^,^^41^

Additional physiological and logistical considerations at high altitude include delayed gastric emptying^68^ and equipment limitations. Flow metres must be recalibrated as they tend to under-read flow rates at high altitude.^7^ Some ventilators deliver tidal volumes lower than set during controlled ventilation owing to decreased gas density and viscosity at high altitude.^7^ Capnographs require altitude-specific calibration, with low *P*co_2_ potentially compromising accuracy.^7^ Tracheal and laryngeal mask airway cuff pressures will increase at high altitude; therefore, removal of air from the cuff or filling with water may be indicated.^46^

The current evidence alongside the findings from this review to give cautious clinical insights is presented in Table 3.^7^^,^^18^^,^^29^, ^30^, ^31^^,^^34^^,^^35^^,^^37^, ^38^, ^39^, ^40^, ^41^^,^^43^^,^^47^, ^48^, ^49^, ^50^, ^51^, ^52^, ^53^, ^54^, ^55^, ^56^, ^57^, ^58^, ^59^, ^60^, ^61^ These should be interpreted with caution.

### Regional anaesthetics

Neuraxial (spinal/epidural) anaesthetics are the preferred in-hospital technique at high altitude, owing to minimal effects on ventilation and lower risk of aspiration.^30^ However, Yang and colleagues^30^ and Aksoy and colleagues^46^ demonstrate longer onset times and higher doses of anaesthetic to achieve successful blockade than at low altitude. Factors affecting intrathecal spread include sex, height, intra-abdominal pressure, and spinal anatomy.^69^ However, CSF volume is considered most significant.^69^ Chronic high altitude theoretically increases cardiac output and cerebral blood flow, resulting in increased CSF volumes,^70^^,^^71^ thus increasing anaesthetic requirements.^69^ However, causation has not been established. The mechanisms are poorly understood, and multiple factors are likely involved.

Bupivacaine is the most used neuraxial agent worldwide, particularly for caesarean section, owing to its long duration of action and relative motor sparing compared with other local anaesthetics.^72^

Ropivacaine has pharmacological properties such as bupivacaine but is associated with reduced motor block in the lower extremities, which may theoretically facilitate the normal progression of labour. This rationale likely influenced its selection in Shokry and colleagues’^31^ study. However, their small study demonstrated no significant advantage of ropivacaine over bupivacaine at high altitude.^32^ Further primary research is required to determine whether any neuraxial agent offers clear superiority in high altitude settings.

Unfortunately, neuraxial techniques are largely impractical in pre-hospital contexts, owing to technical complexity, environmental limitations, and patient transport constraints.^51^ Other regional techniques, such as nerve blocks, have not been studied at high altitude but could offer advantages, including avoidance of sedation, cardiovascular stability, and minimal equipment, providing a rapid and predictable onset of long-lasting pain control.^51^ Nevertheless, these advantages must be balanced with safety as nerve blocks may hamper self-rescue, render a limb unusable, and require advanced training to administer. The concept of field-expedient regional blocks (FERBs)^51^ has been developed at low altitute to address certain administrative issues (Supplementary File 5). High-quality primary research of FERBs at high altitude would be extremely valuable.

### Analgesia/sedation

#### Nitrous oxide

N_2_O has several useful properties as an analgesic: it is self-administered through a hand-held mouthpiece, has rapid onset (<5 min) and offset,^52^ and has an excellent safety profile, notably no respiratory depression.^73^ Those treating the patient are also not exposed to therapeutic concentrations.^74^ A randomised, double-blind, multicentre study has demonstrated its efficacy in pre-hospital settings,^52^ and it is used by ski patrols across Canada, Austria, and the United States,^51^ although specific safety and efficacy data have not been reported.

However, important limitations exist for N_2_O at high altitude. First, the efficacy of inhaled N_2_O is lower at high altitude owing to reduced barometric pressure and the resulting decrease in the partial pressure of a given gas concentration. This is demonstrated by James and colleagues,^33^ Nieto and colleagues,^34^ and Cleaton-Jones and colleagues.^35^ James and colleagues^33^ demonstrated that, at sea level, N_2_O 50% increased pain thresholds by a mean of 71.5% compared with only 40% at 1460 m and only 19% at 3300 m,^33^ suggesting increased concentration requirements. Thus, Cleaton-Jones and colleagues^35^ evaluated the efficacy of increasing concentrations of inhaled N_2_O and found that N_2_O 70% was a safe and effective analgesic at 1610 m.^36^ However, at very high altitudes, the risk of hypoxia, with increasing concentrations in an already oxygen-depleted environment, precludes its use.^75^ However, results are inconsistent between studies. Wood and colleagues^32^ hypothesised that the conversion rate from inhaled N_2_O to another labour analgesic modality would be higher at 1646 m than near sea level, but no statistically significant difference was identified. There may be several factors, in addition to analgesic efficacy, that explain this. Moreover, the data were derived from a retrospective review of records; therefore, a causal relationship between altitude and conversion rates cannot be demonstrated.

Second, N_2_O is also cumbersome to transport, as it is stored in 2- or 5-L steel or composite cylinders, rendering it impractical for most pre-hospital use.^51^

Third, the nature of the pressurised gas mixture reduces the range of ambient temperatures in which it can be used. Direct sunlight and high temperatures increase the risk of cylinder rupture owing to overpressure, and <5°C N_2_O liquefies, posing a problem in high altitude environments.^38^

#### Methoxyflurane

Methoxyflurane is self-administered by inhalation through a light, hand-held device with rapid onset (<5 min)^53^ and offset of action. Changes in cardiovascular, respiratory, and neurological function during administration are not clinically significant,^53^ with the most common side-effects being dizziness, headache, and tiredness, all short-lasting.^53^ Serious adverse effects are rare, but methoxyflurane is contraindicated in patients with hepatic or renal impairment and those with a personal or family history of malignant hyperthermia.^54^^,^^55^

In recent years, methoxyflurane has overtaken the use of N_2_O in the pre-hospital field in Australia and New Zealand.^56^ Although they have equipotent analgesic effects,^56^ as noted, N_2_O is impractical for pre-hospital use whereas methoxyflurane inhalers weigh approximately 100 g. However, it remains unlicensed for use in several countries and in children <18.^57^

At high altitude, methoxyflurane is beneficial as it is unaffected by reductions in barometric pressure.^76^ However, reductions in ambient temperature reduce saturated vapor pressure and therefore the theoretical ability of agents to vaporise, according to Windsor and colleagues.^18^ However, Egger and colleagues^37^ demonstrated no reduction in efficacy at 1362–2666 m with a mean pain score reduction from 7.2 (standard deviation [sd] 1.0) to 2.9 (1.4) after 15 min of 3 ml of inhaled methoxyflurane. Furthermore, no major adverse events or relevant changes in vital signs were observed.^38^ Wilkes and colleagues^47^ assessed the use of methoxyflurane at a higher altitude of 4470 m and still concluded that it provided rapid and effective analgesia without side-effects. Further high-quality, large-scale studies are required to confirm efficacy at higher altitudes.

#### Ketamine

Ketamine is commonly suggested as the analgesic agent of choice in the pre-hospital environment^58^ and at high altitude.^39^^,^^59^ Low-dose ketamine produces “dissociative anaesthesia” with preservation of self-ventilation, which is ideal where advanced airway management is impractical, and severe hypoxia is a significant risk. In addition, when hypovolaemia is present, its positive cardiovascular effects are beneficial compared with other anaesthetic agents.^40^

Maharjan^29^ showed that ketamine can provide safe and effective anaesthesia for 400 children (aged 2–14 yr) undergoing cleft lip and palate surgery in rural Nepal at elevations of 305–3048 m. However, Grocott and colleagues’^39^ case report using ketamine for emergency anaesthesia at 4243 m demonstrated that slow i.v. administration of 0.5 mg/kg, a dose normally used for analgesia at sea level, resulted in deep anaesthesia and apnoea requiring hand ventilation for 5 min. Therefore, careful titration and the knowledge and presence of simple airway and ventilation adjuncts during its use are vital. Bishop and colleagues’^41^ case series of 11 patients undergoing urgent surgery at 3900 m in Nepal demonstrated that 100% of lowland residents (*n*=2), but only 11% of highland residents (*n*=1), required supplemental oxygen administration after their SpO_2_ decreased <80% despite simple airway manoeuvres. No patients required manual ventilation, suggesting that lowland residents may be more susceptible to the effects of ketamine in a hypobaric hypoxic environment than highlanders who are chronically acclimatised.^41^ This inter-individual variability suggests that the “safe” use of ketamine is context dependent.

The use of ketamine at high altitude will continue to be guided by further clinical experience and, ideally, high-quality studies.

#### Fentanyl

Fentanyl has been recommended as the first-line opioid for pre-hospital analgesia as it can provide acute pain relief without the need for IV access when given via intranasal, transmucosal, or sublingual routes.^77^^,^^78^ However, opioids induce hypoxia by diminishing hypoxic ventilatory drive,^60^ and various studies have demonstrated increased sensitivity to opioids after exposure to intermittent hypoxia.^79^, ^80^, ^81^ Therefore, it is hypothesised that opioid requirements are reduced at high altitude. Rabbitts and colleagues’^41^ retrospective observational study of 102 children at 150, 1690, and 3399 m showed that patients at high altitude received 40% less opioid than patients at sea level. However, recommendations on opioid administration cannot be made from these data as it only considers opioid administration rather than requirement. This study serves as hypothesis generating for a future well-designed, adequately powered prospective study to detect a clinically significant difference in opioid requirements between low altitudeand high altitude.

#### Diazepam

Benzodiazepines are commonly used in emergency medicine because of their analgesic, anxiolytic, and sedative effects.^43^ However, ventilatory depression is common, especially when used in conjunction with opioid analgesia.^61^ Röggla and colleagues^42^ reviewed the effect of low-dose (5 mg orally) diazepam on ventilatory response at 171 *vs* 3000 m in lowlanders. At 171 m, 5 mg diazepam had no negative effects on *P*a_O2_. At 3000 m, there was a reduction in respiratory rate, with a consequent significant increase in *P*a_CO2_ (33.3 mm Hg compared with 30.1 in placebo; *P*<0.05) and a decrease in *P*a_O2_ (60.0 mm Hg compared with 66.3 in placebo; *P*<0.005).^43^ This suggests that even small doses of benzodiazepines can inhibit a satisfactory ventilatory response at altitude. Larger, higher-quality studies are needed to strengthen the power of the conclusions by demonstrating reproducibility.

### Local anaesthetics

Local anaesthetics may be an effective method of pre-hospital analgesia; however, only one study exists to inform their use at high altitude. Zhang and colleagues^43^ show that individuals residing at high altitude exhibit significant changes in the pharmacokinetics of lidocaine compared with those at LA. These changes are attributed to a notable decrease in the activity, protein concentration, and mRNA expression of CYP3A4 under chronic high altitude hypoxic conditions.^44^ This has important clinical implications, suggesting that individuals living at HA may require dose adjustment of all medications metabolised by CYP3A4, which includes both lidocaine and benzodiazepines, to ensure therapeutic efficacy and avoid potential toxicity. Further studies are required to demonstrate reproducibility and real-world context, so no clinical insights can be given (Table 3^7^^,^^18^^,^^29^, ^30^, ^31^^,^^34^^,^^35^^,^^37^, ^38^, ^39^, ^40^, ^41^^,^^43^^,^^47^, ^48^, ^49^, ^50^, ^51^, ^52^, ^53^, ^54^, ^55^, ^56^, ^57^, ^58^, ^59^, ^60^, ^61^).

### Clinical insights for use of anaesthetics at high altitude

Clinical insights for use of anaesthetics at high altitudeare presented in Table 3.^7^^,^^18^^,^^29^, ^30^, ^31^^,^^34^^,^^35^^,^^37^, ^38^, ^39^, ^40^, ^41^^,^^43^^,^^47^, ^48^, ^49^, ^50^, ^51^, ^52^, ^53^, ^54^, ^55^, ^56^, ^57^, ^58^, ^59^, ^60^, ^61^

## Strengths and limitations

This scoping review provides a broad and comprehensive assessment of the available literature on anaesthetic administration at high altitude. The use of the Arksey and O’Malley framework and reporting in accordance with PRISMA-ScR enhances methodological transparency and reproducibility. Methodological characteristics were descriptively appraised using CASP, and overall certainty of evidence was characterised using GRADE to support cautious interpretation. Finally, it recommends pertinent areas for future research to better inform future clinical recommendations.

In contrast, the 21 included articles were identified by a single author. Although this process could be perceived as a limitation, it still fulfils the criteria for a rapid scoping strategy.^82^ However, we acknowledge that this review may warrant replication by other groups. The low number of included papers highlights the paucity of research on the effects of high altitude on anaesthetic administration. Small sample sizes and observational or anecdotal methodologies limit power, reproducibility, and robustness of many findings.

Substantial heterogeneity amongst the included articles limits data synthesis; there is no meta-analysis of the included articles. Accordingly, findings should be interpreted as an exploratory mapping of the evidence base rather than definitive guidance.

## Future studies

The included articles demonstrate that high-quality, adequately powered primary research is urgently needed to strengthen the evidence base for HA anaesthetic practice. Robust observational cohorts or pragmatic RCTs with standardised data collection, multicentre collaboration, and altitude-specific methodology represent the most viable strategies. Primary research into FERBs at high altitude would be extremely valuable in the pre-hospital context.

To translate the emerging clinical insights into practice, experts must collaborate to develop formal consensus-based clinical guidelines. Such guidelines should integrate the best available evidence with expert clinical judgement to standardise care and improve safety in pre-hospital high altitude anaesthetic delivery.

## Conclusion

This review highlights the limited evidence for pre-hospital anaesthesia practice at high altitude, despite the increased demand for HA travel and habitation and its inherently high-risk setting. Available studies indicate that HA undoubtedly alters anaesthetic safety and efficacy. However, robust conclusions are hindered by study quality, sample sizes, and poor external validity to pre-hospital environments. Despite these limitations, key insights emerge:

1) Inhalational agents are unreliable at high altitude and impractical for pre-hospital use.
2) TIVA using propofol-fentanyl titrated to depth of anaesthesia can be used safely at high altitude, but requires caution and appropriate monitoring.
3) Ketamine can be used safely at high altitude with airway support available.
4) Neuraxial anaesthesia requires dose adjustment at high altitude but is impractical in pre-hospital settings, supporting investigation of peripheral nerve blocks, including FERBs.
5) Methoxyflurane may be a promising first-line pre-hospital analgesic agent at high altitude owing to its portability, efficacy, and favourable safety profile.
6) Evidence for opioids and benzodiazepines is limited, but evidence suggests increased risk for use in this context owing to their impact on ventilatory drive.

These findings underscore the urgent need for robust, altitude-specific research, particularly in pre-hospital contexts. Until such data exist, clinicians must proceed with caution, adapt practices based on physiological principles, and prioritise simplicity, safety, and airway competence when delivering anaesthesia at high altitude.

## Authors’ contributions

Conceptualisation: OH

Methodology: OH, OT

Investigation: OH

Data curation: OH

Formal analysis: OH

Writing—original draft: OH

Visualisation: OH

Project supervision: OH

Validation: SR

Writing—review and editing: OT

Supervision: OT

## Rights retention

For open access, the author has applied a Creative Commons Attribution (CC BY) licence to any author-accepted manuscript version arising from this submission.

## Data availability statement

All data supporting the findings of this study are available within the paper and its supplementary information.

## Funding

Funding from the National Institute for Health and Care Research (NIHR) Exeter Biomedical Research Centre to OWT.

## Declarations of interest

The authors declare no potential conflicts of interest.
