# Supplementary material for: Evaluation of the impact of high altitude on pre-hospital anaesthetic administration: a scoping review
Source: BJA Open. 2026 Aug 24;19:100643. doi: 10.1016/j.bjao.2026.100643 (PMC13528311; doi:10.1016/j.bjao.2026.100643)
Supplement: Supplementary file 1 [file mmc1.docx]

**Supplementary File 1: population, concept, context table**

**Table S1**: Population, Concept, Context (PCC) framework showing study inclusion and exclusion criteria

| **PCC** | **Inclusion** | **Exclusion** | **Rationale for inclusion / exclusion** | **Limitations related to inclusion / exclusion** |
| --- | --- | --- | --- | --- |
| **Population** | Human participants | Animal studies | The aim of this review is to inform real-world anaesthetic practises derived from human studies. | It is possible that animal studies may provide helpful physiological and pharmacological insights that are not ethically or practically obtainable from human subjects. However, human context is vital to draw meaningful, real-world conclusions. |
| **Concept** | Any type of anaesthetic administration: analgesia/ sedation, local, regional or general anaesthesia | No exclusion for this parameter | This aligns with the primary aim of the scoping review: to provide a comprehensive overview of the existing literature. It allows for evaluation of the potential superiority of one method over another and identifies gaps in current research, informing future guidelines and research priorities. | N/A |
| **Context** | Any publication type | No exclusion for this parameter | Summarises all relevant literature to examine the extent, range and nature of existing research and allow conclusions to be drawn regarding the overall state of research activity and identify gaps for future research. This included “Letters to the editor” as letters are a good way to anecdotally pass on findings in an area where high-quality research is difficult to perform. | N/A |
|  | Articles published in English | Articles published in any other language | Translation of non-English studies requires significant time, language expertise and/or financial resources, which are not available for this review.  Translation may also risk misinterpretation or omission of critical information. | This may introduce language bias and potentially exclude relevant data. However, the Cochrane Handbook suggests this impact may be minimal due to the predominance of English in scientific publishing^1^. |
|  | Articles published after 1980 | Articles published prior to 1980 | This ensures relevance to both modern anaesthetic practise (the introduction and widespread adoption of propofol, which is now the most used agent in the UK, occurred in the 80s and 90s^2^) and coincides with the popularisation and commercialisation of high-altitude adventure travel. | This may potentially exclude some pioneering studies published prior to 1980. However, studies post-1980 are more likely to be methodologically robust, aligning with modern standards of evidence-based practise, which can better help to inform current clinical practise and research. |
|  | Any geographical location over >1,500m, whether this is real or simulated altitude | Articles examining anaesthetic administration <1,500m | Physiological adaptations begin to occur ~1,500m^3-5^, which may impact anaesthetic pharmacodynamics. The lowest altitude parameter is used to ensure important data are not excluded.  Simulated settings allow researchers to effectively isolate the effects of altitude without external environmental variability. | N/A |

**Supplementary File 2: search concepts tables**

**Table S2.1:** MEDLINE search concepts table

| **High Altitude** | **Anaesthetic administration** |
| --- | --- |
| MeSH terms:  exp *Altitude /  exp *mountaineering /  Keywords:  Environmental hypoxia  Environmental anoxia  Environmental oxygen deficiency | MeSH terms:  Exp *Anesthetics/  Exp *Anesthesia/  Exp *Prilocaine /  Exp *Lidocaine /  Exp *Bupivacaine /  Exp *Propofol /  Exp *Thiopental /  Exp *Isoflurane /  Exp *Sevoflurane /  Exp *Desflurane /  Exp *Nitrous oxide /  Exp *Midazolam /  Exp *Ketamine /  Exp *Fentanyl /  Exp *Alfentanil /  Exp *Rocuronium /  Exp *Succinylcholine /  Exp *Atracurium /  Exp *Vecuronium bromide / |

**Table S2.2:** EMBASE search concepts table

| **High Altitude** | **Anaesthetic administration** |
| --- | --- |
| Subject headings:  exp *Altitude /  exp *mountaineering /  Keywords:  Environmental hypoxia  Environmental hypoxemia  Environmental anoxia  Environmental oxygen deficiency | Subject headings:  Exp *Anesthetic agent /  Exp *Prilocaine /  Exp *Lidocaine /  Exp *Bupivacaine /  Exp *Propofol /  Exp *Thiopental /  Exp *Isoflurane /  Exp *Sevoflurane /  Exp *Desflurance /  Exp *Nitrous oxide /  Exp *Midazolam /  Exp *Ketamine /  Exp *Fentanyl /  Exp *Alfentanil /  Exp *Rocuronium /  Exp *Suxemethonium /  Exp *Atracurium besilate /  Exp *Vecuronium / |

**Table S2.3:** Web of Science search concepts table

| **High Altitude** | **Anaesthetic administration** |
| --- | --- |
| Key words:  Altitude  Mountain*  Environmental hypox*  Environmental anoxia  Environmental oxygen deficiency | Key words:  Anaesthet*  Anesthe*  Prilocaine, Citanest  Lidocaine, Lignocaine, LMX 4, Versatis, Xylocaine  Bupivacaine, Marcain  Propofol, Diprivan  Thiopental, Thiopentone, Pentothal  Isoflurane, Forane, Sedaconda, Isospire, AErrane  Sevoflurane, Sevorane, Sojourn, Ultane  Desflurane, Suprane,  Nitrous oxide, Entonox, Nitronox, Alnox, (Penthrox)  Midazolam, Versed, Nayzilam, Seizalam  Ketamine, Ketalar, Ketaset, Ketajet, Ketavet, Vetamine, Vetaket  Fentanyl, Abstral, Effentora, Instanyl, PecFent  Alfentanil, Rapifen, Alfenta  Rocuronium, Zemuron, Esmeron  Suxemethonium, Scoline, Sucostrin, Succinylcholine, Anectine, Quelicin  Atracurium, Tracrium, Acurium  Vecuronium, Norcuron |

**Supplementary File 3: data charting**

**Table S3**: Studies included in scoping review

| Title | Authors | Article type | Year | Study numbers | Study location | Study Aims | Sample Population | Level of Altitude  metres (m) | Anaesthetic Sub- category | Anaesthetic Agent(s) | Clinical Recommendations / Conclusions |
| --- | --- | --- | --- | --- | --- | --- | --- | --- | --- | --- | --- |
| The Median Effective Dose of Intrathecal Hyperbaric Bupivacaine for Cesarean Section at Moderately High-Altitude.^6^ | Yang, C. Dong, W. Luo, J. Ma, M. Gao, Y. Ni, L. et al. | Up-down sequential Study | 2024 | 30 | Yunnan Province, China | To determine the median effective dose of bupivaine for spinal anaesthesia at high-altitude | Highlanders | 2000 | Spinal | Bupivacaine | The ED50 of 0.75% hyperbaric bupivacaine at 2,000m is 8.23mg (95% CI, 6.52-9.32mg), which significantly exceeds the ED50 of patients at LA (3.4mg). This is possibly due to the increase in CSF volume at HA. Further studies are required to determine ED90/95 to offer enhanced guidance for clinical practise. |
| Multicenter Study Evaluating Nitrous Oxide Use for Labor Analgesia at High- and Low-Altitude Institutions^7^ | Wood, C. Arbet, J. Amura, C.R. Nodine, P. Collins, MR. Orlando, BS. Myer, DC. et al. | Retrospective observational study | 2022 | 1,856 | Colorado, New York, North Carolina and Nashville, USA | To compare the efficacy of nitrous oxide analgesia in labour at low vs high altitude and the odds of converting from nitrous oxide to other labour analgesics. | Both - comparison study | Near sea-  level vs 1646m | Analgesia / sedation | Nitrous oxide | The odds of converting from 50% N2O to another analgesic modality had no statistically significant difference between high versus low altitude institutions (OR = 2.19; 95% CI 1.14-4.21). Therefore, N_2_O is as effective at HA as at sea level. There are significant benefits to the use of nitrous oxide analgesia, and this modality should be available to the millions of women who labour at higher altitudes. |
| Prehospital use of ketamine in mountain rescue: a letter^8^ | Ellerton, J. Paal, P. Brugger, H. | A letter to the editor | 2009 | - | - | To propose some additional considerations from original paper | - | - | Analgesia / sedation | Ketamine | Ketamine is a useful drug in the pre-hospital setting (in the absence of traumatic brain injury). However, the amount of intranasal S-ketamine swallowed leads to variation in effect. Caution is required brining Ketamine across borders. |
| Pharmacokinetics of Lidocaine Hydrochloride Metabolized by CYP3A4 in Chinese Han Volunteers Living at Low Altitude and in Native Han and Tibetan Chinese Volunteers Living at High Altitude.^9^ | Zhang, J. Zhub, J. Yao, X. Duan, Y. Zhou, X. Yang, M. | Unblinded interventional study | 2016 | 53 | Xi'an and Qinghai-Tibetan area, China | To investigate the pharmacokinetics of lidocaine at low vs high altitude in different ethnic populations (Chinese Han volunteers living at low altitude) and in native Han and Tibetan Chinese volunteers living at high altitude | Both - comparison study | 400m (low group), 2200-4500 (high group) | Local | Lidocaine | The t1/2 of lidocaine was 29.8% higher in high altitude versus low altitude groups. This is possibly due to significant decreases in the activity of protein and mRNA expression of CYP3A, as shown in rats. Patients living or travelling to high altitude should be closely monitored, and the dosages of some drugs including lidocaine metabolised by CYP3A1 should be reduced. |
| Spinal anaesthesia at low and moderately high altitudes: a comparison of anaesthetic parameters and hemodynamic changes.^10^ | Aksoy, M. Ince, Ilker. Ahiskalioglu, A. Karaca, O. Bayar, F. Erdem, AF. | Prospective controlled comparative study | 2015 | 155 | Sakarya and Erzurum, Turkey | To determine the differences between highlanders and lowlanders in terms of anaesthetic parameters under neuraxial anaesthesia | Both - comparison study | 31m (low group), 1890m (high group) | Spinal | Bupivacaine | Onset of action of 0.5% hyperbaric bupivacaine was significantly shorter at sea-level (14.34 +/- 0.88 minutes) than at 1,890m (20.38 +/- 1.46). Duration of both the motor and sensory block was also longer at sea-level (310.2 +/-104.2 and 200.2+/-50) than at 1,890m (200.4 +/- 103.2 and 155.2 +/- 60.7). This is possibly due to increased CSF volume at HA. Further studies are required to demonstrate causality. |
| Perioperative opioid requirements are decreased in hypoxic children living at altitude.^11^ | Rabbitts, JA. Croenewald, CB. Dietz, NM. Morales, C. Rasanen, J. | Retrospective observational study | 2010 | 271 | Lima and Cusco, Peru | To investigate the effect of altitude on perioperative Fentanyl requirement in children undergoing cleft lip / palate surgery | Both - comparison study | 150m (low group), 3399m (high group) | Analgesia / sedation | Fentanyl | Patients at high altitude received 40% less opioid compared to patients at sea level. Whether this difference is due to altitude or altitude-induced hypoxia, requires further study. |
| Effects of intrapartum epidural analgesia at high altitudes: maternal, fetal, and neonatal outcomes. A randomized controlled trial of two formulations of analgesics.^12^ | Shokry, M. Manaa, ES. Shoukry, RA. Shokier, MH. Elsedfy, GO. El-Aziz, AE-S. | Prospective randomised controlled study | 2010 | 80 | Aseer region, Saudi Arabia | To investigate whether intrapartum epidural bupivacaine is superior over ropivacaine at high altitude or if either medication has an influence on mothers, foetuses or newborns | Highlanders | 2200m | Epidural | Bupivacaine, ropivacaine | There is no advantage for epidural Ropivacaine 0.2% over Bupivacaine 0.125% in addition to Fentanyl for labour analgesia at HA (2,200m), and no harmful effects were found on mothers, foetuses or newborns. |
| Methoxyflurane as an analgesic for prehospital use at high altitude.^13^ | Windsor, J. Van der Kaail, J. Ellerton, J. Oxer, H. Hillebrant, D. Rodway, G. | A letter to the editor | 2009 | - | - | To propose further study into the use of Penthrox in the pre-hospital, high altitude environment | - | - | Analgesia / sedation | Methoxyflurane (Penthrox) | Methoxyflurane is an effective source of analgesia. Especially useful in the pre-hospital setting due to its portability and easy use. However, reduction in barometric pressure and temperature reduces saturated vapour pressure, so theoretically the ability of Penthrox to vaporise at altitude. |
| Propofol-fentanyl anaesthesia at high altitude: anaesthetic requirements and haemodynamic variations when compared with anaesthesia at low altitude.^14^ | Puri, GD. Jayant, A. Dorje, M. Tashi, M. | Prospective controlled interventional study | 2008 | 30 | Chandigarh and Leh, India | To demonstrate the safety of propofol-fentanyl anaesthesia at high altitude titrated to bispectral index (BIS) (3505m above sea level) in native highlanders | Both - comparison study | 304m (low group), 3505m (high group) | General | Propofol-fentanyl | High-altitude dwellers required significantly higher doses of anaesthesia at induction (2.32 +/- 0.64 vs. 1.41 +/- 0.24mg/kg, P<0.0001) and maintenance (6.22 +/-1.14 vs 4.61 +/- 1/29 mg/kg/h, P<0.01) compared to lowlanders. However, propofol-fentanyl anaesthesia, titrated to BIS, is safe at HA. |
| Ketamine for emergency anaesthesia at very high altitude (4243m above sea-level).^15^ | Grocott, MPW. Johannson, L. | Case report | 2007 | 1 | Pheriche, Nepal | To describe the successful use of ketamine as anaesthesia at very high altitude. | Highlanders | 4232m | Analgesia / sedation | Ketamine | Ketamine can be successfully used as a general anaesthetic agent at very HA (4243m) in a patient with haemorrhagic shock. However, significantly lower doses (approx ¼) are required than at sea-level to produce a deep anaesthetic effect, requiring ventilation. Careful titration is strongly advised, alongside the availability of airway equipment and the skills to use them. |
| Anaesthesia for cleft lip surgery: a challenge in rural Nepal.^16^ | Maharjan, SK | Retrospective observational study | 2003 | 400 | Himalaya region, Nepal | To study the safety of different anaesthetic procedures for children with cleft lip / palate procedures in rural Nepal | Both - comparison study | 305 - 3,050m | General | Ketamine + Midazolam OR ketamine + halothane | Oral ketamine premedication and intramuscular or intravenous boluses of ketamine with atropine can be used without intubation for simple cleft lip surgery in children over one year of age, with oxygen saturation monitoring and all equipment for intubation and ventilation should be ready in hand. |
| Ketamine anesthesia at high altitude.^17^ | Bishop, RA. Litch, JA. Stanton, JM. | Case series | 2000 | 11 | Himalaya region, Nepal | To assess the safety and efficacy of the use of ketamine at high altitude and in remote areas | Highlanders | 3,900m | Analgesia / sedation | Ketamine | Ketamine offers a safe and effective means of anaesthetise at very high altitude (3,900m) without the need for specialist equipment or training, by careful clinicians experienced in basic airway management. |
| Effect of low dose sedation with diazepam on ventilatory response at moderate altitude.^18^ | Roggla, G. Roggla, M. Wagner, A. Seidler, D. Podolsky. | Randomised, double blind, placebo-controlled crossover trial | 1994 | 8 | Alps, Austria | To evaluate the effect of low dose benzodiazepines on ventilatory response at moderate altitude | Lowlanders | 171m (low group), 3,000m (high group) | Analgesia / sedation | Diazepam | At 171m low dose Diazepam (5mg PO) had no negative effects on PaO_2_. At 3,000m there was a reduction in respiratory rate with a consequent significant rise in PaCO_2_ (33.3mmHg compared to 30.1 in placebo, p<0.05) and fall in PaO_2_ (60.0mmHg compared to 66.3 in placebo, p<0.005) |
| Closed circuit anesthesia is accurate at any altitude^19^. | Baumgarten, RK. | A letter to the editor | 1985 | - | - | A response to “anaesthetic considerations at moderate altitude”, which reviewed the effects of altitude on the delivery of anaesthetics and highlighted an inadequacy of the minimum alveolar concentration concept. | - | - | General | Halothane | The inadequacy of MAC at altitude can be overcome with use of closed-circuit anaesthesia. |
| Frequency of anesthetic overdose with mean alveolar concentration-guided anesthesia at high altitude^20^. | Giraldo, JC. Acosta, C. Giraldo-Grueso, M. | Prospective cohort study | 2019 | 50 | Bogota, Colombia | To examine the frequency of anaesthetic overdose measured with bispectral index at high altitude | Highlanders | 2,600m | General | Sevoflurane | Anaesthetic overdose with adequate MAC was higher than expected at 2,600m. BIS-guided anaesthesia is a more reliable tool at altitude. |
| Minimum alveolar concentration based anesthesia in high altitude and anesthetic overdose: result needs cautious acceptance^21^. | Karim, HMR | Letter to the editor | 2019 | - | - | A response to "Frequency of anaesthetic overdose with mean alveolar concentration-guided anaesthesia at high altitude" | - | - | General | Sevoflurane | MAC value depends on several parameters alongside altitude. Each decade of age affects MAC by 6%. Therefore, further study is required to demonstrate the relationship between altitude and MACage. |
| Nitrous oxide analgesia and altitude^22^. | James, MFM. Manson, EDM. Dennett, JE. | Prospective controlled interventional study | 1982 | 20 | Salisbury, Zimbabwe | To study the influence of altered barometric pressure on the degree of analgesia produced by 50% N_2_0 using the same subjects and observers at each pressure level | Native lowlanders | 0m (low group), 1460m (medium group), 3,300m (high group). NB. Simulated altitudes | Analgesia / sedation | Nitrous oxide | The difference between the analgesic effects of inhaled 50% N_2_0 was at increasing altitudes is statistically significant. At sea level, 50% N_2_0 increased pain thresholds by a mean of 71.5%, at 1460m by 40% and at 3300m by only 19%. |
| Nitrous oxide at higher elevation^23^. | Nieto, JM. Rosen, P. | Prospective cohort study | 1980 | 124 | Colarado, USA | To determine whether the use of 70% N_2_0 with 30% O_2_ would be a safe and effective alternative to 50% N_2_0 at 1610m elevation | Highlanders | 1,610m | Analgesia / sedation | Nitrous oxide | The mixture of 70% N_2_0 with 30% O_2_ was safe and effective in treating juvenile and young adult patients with pain in an ED at 1,610m. |
| Clinical effects of nitrous oxide and oxygen mixtures at sea-level and at 1700 metres altiude^24^. | Cleaton-Jones, P. Moyes, DG. Whittaker, AM. | Prospective controlled interventional study | 1980 | 52 | Stellenbosh, South Africa | To examine the subjective and objective reactions to the inhalation of 50%, 60% and 70% mixtures of N_2_0 at sea level at 1,700m | Native lowlanders | 0m (low group), 1,700m (high group) | Analgesia / sedation | Nitrous oxide | Only marginal differences were shown between the effects of 50%, 60% and 70% inhaled N_2_0 at sea-level compared to 1,700m. |
| Methoxyflurane for procedural analgesia at 4470m altitude^25^. | Wilkes, M. Heath, EC. Mason, NP. | Case report | 2018 | 1 | Machermo, Nepal | A case report to promote the use of Methoxyflurane (Penthrox) at high altitude (2,270m) | Native lowlanders | 2,270m | Analgesia / sedation | Methoxyflurane (Penthrox) | Methoxyflurane provided rapid, effective analgesia for a single patient at 4470m altitude without any complication. |
| Efficacy and safety of methoxyflurane for treatment of acute traumatic pain by EMTs during Alpine Rescue Operations: The"PainDrop" trial^26^ | Egger, A. Huber, T. Heschl, S. Fiegl, J. Burger, J. Trimmel, H. et al | Prospective observational study | 2022 | 20 | Alps, Austria | To evaluate the efficacy and safety of methoxyflurane for the treatment of acute traumatic pain by EMTs during alpine rescue operations | Native lowlanders | 1,362m - 2,666m | Analgesia / sedation | Methoxyflurane (Penthrox) | Methoxyflurane shows no reduction in efficacy at moderate altitude (1,362m-2,666m). The mean initial NRS pain scale was 7.2 (SD 1.0), which significant reduced to 2.9 after 15 minutes of 3ml of inhaled methoxyflurane use (95% CI 2.2-3.6; SD 1.4, p<0.001). No major adverse events or relevant changes in vital signs were observed. |

*ED50 = effective dose in 50% of the population; CI = confidence interval; LA = low altitude; CSF = cerebrospinal fluid; HA = high altitude; ED90/95% = effective dose in 90/95% of the population; N_2_O = nitrous oxide; t1/2 = half-life; BIS = bispectural index; PO = by mouth; PaCO_2_ = the partial pressure of carbon dioxide in arterial blood; PaO_2_ = the partial pressure of oxygen in arterial blood; MAC = mean alveolar concentration; ED = Emergency Department; EMTs = Emergency Medical Teams; NRS = numeric rating scale; SD = standard deviation.*

**Supplementary File 4: Critical appraisal**

**Table S4:** **CASP critical appraisal of the included articles**

| **Study** | **Study Design** | **CASP checklist used** | **Positive / Methodologically sound** | **Negative / Relatively poor methodology** | **Unknowns / Unclear reporting** |
| --- | --- | --- | --- | --- | --- |
| Yang et al, 2015^6^ | Up-down sequential study | CASP RCT (dose-finding trial appraisal) | Clear aim; appropriate sequential dose-finding approach; objective endpoint (ED50). | Single setting limits generalisability; does not address comparative effectiveness. | Randomisation/allocation concealment and blinding typically not applicable/unclear for up–down designs; sample size justification unclear. |
| Wood et al, 2022^7^ | Retrospective observational study | CASP Cohort | Large multicentre dataset; clinically relevant outcomes; real-world practice settings. | Retrospective design; confounding by site/patient differences likely; exposure (altitude) may proxy other factors. | Adjustment strategy, missing data handling, and outcome assessment methods not fully specified in extraction. |
| Ellerton et al, 2009^8^ | A letter to the editor | No CASP checklist available (non-empirical) | Clinically relevant observations; highlights feasibility considerations. | Not a systematic study; no defined methods; high risk of selection/reporting bias. | Case ascertainment, denominator, and outcomes not described. |
| Zhang et al, 2016^9^ | Unblinded interventional study | CASP Cohort | Objective PK endpoints; defined comparison groups (altitude/ethnicity); biologically plausible mechanism. | Limited clinical outcomes; potential selection bias; small samples typical for PK studies. | Matching/adjustment for confounders (diet, co-meds, acclimatisation) unclear; assay blinding/QC not described in extraction. |
| Aksoy etc al, 2015^10^ | Prospective controlled comparative study | CASP Cohort | Direct comparison; clinically relevant haemodynamic outcomes; practical anaesthesia parameters. | Non-randomised; baseline differences may drive effects; altitude may correlate with other care differences. | Participant selection, missing data, and confounder control not clearly reported in extraction. |
| Rabbitts et al, 2010^11^ | Retrospective observational study | CASP Cohort | Clear clinical outcome; relevant population; plausible physiological rationale (hypoxia). | Confounding (case mix, surgery type, analgesic protocols) likely; observational design. | How opioid requirements were standardised and adjusted is unclear; follow-up window not specified in extraction. |
| Shokry et al, 2010^12^ | Prospective randomised controlled trial | CASP RCT | Randomised design; maternal/fetal/neonatal outcomes; comparative formulation question. | Risk of performance/detection bias if blinding incomplete; single setting may limit generalisability. | Randomisation method, allocation concealment, blinding, and attrition details not in extraction. |
| Windsor et al, 2009^13^ | A letter to the editor | CASP Cohort | Highly pragmatic; relevant to austere/altitude settings; focuses on feasibility/analgesia. | Likely small, non-randomised; confounding by injury severity and co-interventions. | Outcome measurement (pain scoring timing, adverse event capture) unclear in extraction. |
| Puri et al, 2008^14^ | Prospective controlled interventional study | CASP Cohort | Direct low vs high altitude comparison; objective haemodynamic outcomes; clinically relevant dosing/requirements. | Non-randomised; protocol differences across sites possible; selection bias. | Standardisation of anaesthesia technique and monitoring across sites unclear in extraction. |
| Grocott et al, 2007^15^ | Case report | No CASP checklist available (case report/series) | High relevance to extreme altitude; detailed clinical feasibility insights likely. | No comparator; cannot estimate effect size; high susceptibility to publication bias. | Denominator and adverse event ascertainment unclear. |
| Maharjan, 2003^16^ | Retrospective observational study | No CASP checklist available (case series/service description) | High contextual relevance; describes implementation challenges and pragmatic adaptations. | Non-comparative; limited internal validity; outcomes may be selectively reported. | Case selection criteria and follow-up not specified in extraction. |
| Bishop et al, 2000^17^ | Case series | No CASP checklist available (case series) | Clinically relevant; contributes early evidence for feasibility/safety. | No control group; bias likely; limited generalisability. | Methodological detail (sampling, outcomes, adverse events) unclear in extraction. |
| Roggla et al, 1994^18^ | Randomised, double blind, placebo-controlled crossover trial | CASP RCT (experimental trial appraisal) | Objective physiological outcome; controlled exposure; mechanistic insight. | May be small and highly selected; limited clinical endpoints. | Randomisation/crossover methods, blinding, and washout details not in extraction. |
| Baumgarten, 1985^19^ | A letter to the editor | No CASP checklist available (technical validation) | Direct relevance to equipment performance; focused technical question. | Not a clinical outcomes study; external validity depends on testing conditions. | Validation standards, test conditions, and error quantification unclear in extraction. |
| Giraldo et al, 2019^20^ | Prospective cohort study | CASP Cohort | Clinically important safety outcome; practice-relevant; likely real-world denominator. | Confounding by case mix and protocol adherence; observational bias. | Definitions of “overdose”, ascertainment, and adjustment methods unclear in extraction. |
| Karim, 2019 | A letter to the editor | No CASP checklist available (non-empirical) | Critical contextual interpretation; highlights limitations and risk of over-interpretation. | Not an empirical study; cannot quantify risk or causality. | Evidence base and methods for critique not specified. |
| James et al, 1982^22^ | Prospective controlled interventional study | No CASP checklist available (non-empirical/brief) | Raises clinically relevant concern; may summarise observations. | Likely lacks methods and systematic data collection. | Population, outcomes, and context not clear. |
| Nieto et al, 1980^23^ | Prospective cohort study | CASP Cohort (if observational) OR No CASP (if letter) | Clinically relevant topic; potential early comparative insight. | Older design standards; likely small sample; limited control of confounding. | Study design and methods unclear from extraction alone. |
| Cleaton-Jones et al, 1980^24^ | Prospective controlled interventional study | CASP RCT (experimental trial appraisal) | Direct comparison across altitudes; objective physiological/clinical effects. | Possible small sample; older reporting standards; limited modern monitoring. | Randomisation/blinding and adverse event capture unclear in extraction. |
| Wilkes et al, 2018^25^ | Case report | No CASP checklist available (case report/series) | Extreme-altitude relevance; pragmatic procedural analgesia focus. | No comparator; limited generalisability; publication bias likely. | Systematic adverse event follow-up unclear. |
| Egger et al, 2022^26^ | Prospective observational study | CASP Cohort | Prospective design; operational alpine setting; includes safety outcomes. | Not randomised; confounding by injury severity/co-interventions. | Comparator definition, missing data handling, and longer-term outcomes unclear in extraction. |

**Supplementary File 5: Field-expedient regional blocks (FERBs)**

**Table S5**: The concept of field-expedient regional blocks (FERBs)^28^

FERBs refer to nerve block techniques that:

• Have consistent approaches using reliable anatomical landmarks;

• Can be performed reliably with a predictable distribution of effect;

• Avoid structures that carry high risk or consequences of injury (eg, pleura or major vessels);

• Have a low risk of unwanted effects (eg, phrenic nerve block and diaphragmatic paralysis, which frequently accompanies interscalene brachial plexus block);

• Require drug volumes that are well within the maximum safe dose of the selected LA;

• Tend toward slower drug absorption, reducing the risk of systemic toxicity;

• Are easily learned and can be practiced by austere medicine practitioners working in a variety of settings to become proficient before using them in the field;

• Do not require highly specialized equipment.

*FERB = field-expedient regional block; LA = local anaesthetic.*

**References**

1.	Higgins J, Green S. Cochrane Handbook for Systematic Reviews of Interventions Version 5.1.0 (updated March 2011): The Cochrane Collaberation 2011 [Available from: <https://training.cochrane.org/handbook/archive/v5.0.0/chapter_10/10_2_2_4_language_bias.htm>.

2.	Committee JF. Anaesthesia - general London: BMJ Group and Pharmaceutical Press; 2025 [Available from: <https://bnf.nice.org.uk/treatment-summaries/anaesthesia-general/>.

3.	Leissner KB, Mahmood FU. Physiology and pathophysiology at high altitude: considerations for the anesthesiologist. Journal of Anesthesia. 2009;23(4):543-53.

4.	CPG) JTSCPGJ. Altitude Emergencies in the Prehospital Environment 2024 [Available from: <https://jts.health.mil/assets/docs/cpgs/Altitude_Emergencies_Prehospital_Environment_05_Mar_2024_ID95_v1.1.pdf>.

5.	Brown JPR, Grocott MPW. Humans at altitude: physiology and pathophysiology. Continuing Education in Anaesthesia, Critical Care and Pain. 2013;13(1):17-22.

6.	Yang C, Dong W, Luo J, Ma M, Gao Y, Ni L, et al. The Median Effective Dose of Intrathecal Hyperbaric Bupivacaine for Cesarean Section at Moderately High-Altitude. Drug Des Devel Ther. 2024;18:1313-9.

7.	Wood C, Arbet J, Amura CR, Nodine P, Collins MR, Orlando BS, et al. Multicenter Study Evaluating Nitrous Oxide Use for Labor Analgesia at High- and Low-Altitude Institutions. Anesth Analg. 2022;134(2):294-302.

8.	Ellerton J, Paal P, Brugger H. Prehospital use of ketamine in mountain rescue. Emergency Medicine Journal. 2009;26(10):760-1.

9.	Zhang J, Zhu J, Yao X, Duan Y, Zhou X, Yang M, et al. Pharmacokinetics of Lidocaine Hydrochloride Metabolized by CYP3A4 in Chinese Han Volunteers Living at Low Altitude and in Native Han and Tibetan Chinese Volunteers Living at High Altitude. Pharmacology. 2016;97(3-4):107-13.

10.	Aksoy M, Ince I, Ahıskalıoglu A, Karaca O, Bayar F, Erdem AF. Spinal anaesthesia at low and moderately high altitudes: a comparison of anaesthetic parameters and hemodynamic changes. BMC Anesthesiology. 2015;15(1):123.

11.	Rabbitts JA, Groenewald CB, Dietz NM, Morales C, Räsänen J. Perioperative opioid requirements are decreased in hypoxic children living at altitude. Paediatr Anaesth. 2010;20(12):1078-83.

12.	Shokry M, Manaa EM, Shoukry RA, Shokeir MH, Elsedfy GO, Abd El-Aziz Ael S. Effects of intrapartum epidural analgesia at high altitudes: maternal, fetal, and neonatal outcomes. A randomized controlled trial of two formulations of analgesics. Acta Obstet Gynecol Scand. 2010;89(7):909-15.

13.	Windsor J, van der Kaaij J, Ellerton J, Oxer H, Hillebrandt D, Rodway G. Methoxyflurane as an analgesic for prehospital use at high altitude. High Alt Med Biol. 2009;10(2):201-2.

14.	PURI GD, JAYANT A, DORJE M, TASHI M. Propofol–fentanyl anaesthesia at high altitude: anaesthetic requirements and haemodynamic variations when compared with anaesthesia at low altitude. Acta Anaesthesiologica Scandinavica. 2008;52(3):427-31.

15.	Grocott MP, Johannson L. Ketamine for emergency anaesthesia at very high altitude (4243 m above sea-level). Anaesthesia. 2007;62(9):959-62.

16.	Maharjan SK. Anaesthesia for cleft lip surgery--challenge in rural Nepal. Kathmandu Univ Med J (KUMJ). 2004;2(2):89-95.

17.	Bishop RA, Litch JA, Stanton JM. Ketamine anesthesia at high altitude. High Alt Med Biol. 2000;1(2):111-4.

18.	Röggla G, Röggla M, Wagner A, Seidler D, Podolsky A. Effect of low dose sedation with diazepam on ventilatory response at moderate altitude. Wien Klin Wochenschr. 1994;106(20):649-51.

19.	Baumgarten RK. Closed Circuit Anesthesia is Accurate at any Altitude. Anesthesia & Analgesia. 1985;64(8):843.

20.	Giraldo JC, Acosta C, Giraldo-Grueso M. Frequency of anesthetic overdose with mean alveolar concentration-guided anesthesia at high altitude. Medical Gas Research. 2018;8(4):150-3.

21.	Reazaul Karim HM. Minimum alveolar concentration based anesthesia in high altitude and anesthetic overdose: result needs cautious acceptance. Med Gas Res. 2019;9(2):107.

22.	James MF, Manson ED, Dennett JE. Nitrous oxide analgesia and altitude. Anaesthesia. 1982;37(3):285-8.

23.	Nieto JM, Rosen P. Nitrous oxide at higher elevations. Annals of Emergency Medicine 1980;9:610-2.

24.	CLEATON-JONES P, MOYES DG, WHITTAKER AM. Clinical effects of nitrous oxide and oxygen mixtures at sea-level and at 1700 metres altitude. Anaesthesia. 1979;34(9):859-62.

25.	Wilkes M, Heath EC, Mason NP. Methoxyflurane for Procedural Analgesia at 4470 m Altitude. Wilderness & Environmental Medicine. 2018;29(3):388-91.

26.	Egger A, Huber T, Heschl S, Fiegl J, Burger J, Trimmel H, et al. Efficacy and Safety of Methoxyflurane for Treatment of Acute Traumatic Pain by EMTs during Alpine Rescue Operations: The "PainDrop" Trial. Prehosp Emerg Care. 2023;27(8):987-92.

27.	Group GW. GRADE Handbook [Internet] 2013. Available from: <https://gdt.gradepro.org/app/handbook/handbook.html#h.qmsmbyw3yxsl>.

28.	Fink PB, Wheeler AR, Smith WR, Brant-Zawadzki G, Lieberman JR, McIntosh SE, et al. Wilderness Medical Society Clinical Practice Guidelines for the Treatment of Acute Pain in Austere Environments: 2024 Update. Wilderness & Environmental Medicine. 2024;35(2):198-218.
